# Survival and Cost-Effectiveness of Trabectedin Compared to Ifosfamide Monotherapy in Advanced Soft Tissue Sarcoma Patients

**DOI:** 10.1155/2019/3234205

**Published:** 2019-06-02

**Authors:** Michiel C. Verboom, Hans Gelderblom, J. Martijn Kerst, Neeltje Steeghs, Anna K. L. Reyners, Stefan Sleijfer, Winette T. A. van der Graaf, Wilbert B. van den Hout

**Affiliations:** ^1^Department of Medical Oncology, Leiden University Medical Center, P.O. Box 9600, 2300 RC Leiden, Netherlands; ^2^Department of Medical Oncology and Clinical Pharmacology, Netherlands Cancer Institute-Antoni van Leeuwenhoek, P.O. Box 90203, 1006 BE Amsterdam, Netherlands; ^3^Department of Medical Oncology, University Medical Center Groningen, University of Groningen, P.O. Box 30001, 9700 RB Groningen, Netherlands; ^4^Department of Medical Oncology, Erasmus MC Cancer Institute, P.O. Box 5201, 3008 AE Rotterdam, Netherlands; ^5^Department of Medical Oncology, Radboud University Medical Center, P.O. Box 9101, 6500 HB Nijmegen, Netherlands; ^6^Department of Medical Decision Making, Leiden University Medical Center, P.O. Box 9600, 2300 RC Leiden, Netherlands

## Abstract

Trabectedin and ifosfamide are among the few cytostatic agents active in advanced soft tissue sarcomas (STSs). Trabectedin is most potent against so-called L-sarcomas (leiomyosarcoma and liposarcoma). The survival gain and cost-effectiveness of these agents in a second-line setting were analysed in the setting of advanced STS after failure of anthracyclines. A prospective observational trial had previously been performed to assess the use of trabectedin in a Dutch real-world setting. Data on ifosfamide monotherapy were acquired from previous studies, and an indirect comparison of survival was made. A state-transition economic model was constructed, in which patients could be in mutually exclusive states of being preprogression, postprogression, or deceased. The costs and quality-adjusted life years (QALYs) for both treatments were assessed from a Dutch health-care perspective. Separate analyses for the group of L-sarcomas and non-L-sarcomas were performed. Trabectedin treatment resulted in a median progression-free survival of 5.2 months for L-sarcoma patients, 2.0 months for non-L-sarcoma patients, and a median overall survival of 11.8 and 6.0 months, respectively. For L-sarcoma patients, trabectedin offered an increase of 0.368 life years and 0.251 QALYs compared to ifosfamide and €20,082 in additional costs, for an incremental cost-effectiveness ratio (ICER) of €80,000 per QALY gained. In the non-L-sarcoma patients, trabectedin resulted in 0.413 less life years and 0.266 less QALYs, at the increased cost of €4,698. The difference in survival between drugs and the acquisition costs of trabectedin were the main influences in these models. Trabectedin was shown to have antitumour efficacy in advanced L-sarcoma. From a health economics perspective, the costs per QALY gained compared to ifosfamide monotherapy that may be acceptable, considering what is currently regarded as acceptable in the Netherlands.

## 1. Introduction

Soft tissue sarcomas (STSs) are a rare group of malignancies arising from mesenchymal cells comprising one percent of all adult malignancies. STSs in general are relatively insensitive to chemotherapy compared to tumours of epithelial origin. Some drugs, such as doxorubicin, have been found active in a range of different sarcoma subtypes, whereas others show only activity in specific subtypes, such as crizotinib in the inflammatory myofibroblastic tumour [1]. Trabectedin is a drug active in several subtypes, with most notable effect in leiomyosarcoma and liposarcoma. It has a unique mechanism of action in binding to the minor groove of DNA and also in influencing the tumour environment [2, 3].

Trabectedin was approved for clinical use in Europe in 2007 for patients with advanced STS after failure to anthracyclines and ifosfamide or for patients unsuited to receive these agents. At this time, studies with a randomised comparison with other treatment options were not available. Therefore, before market authorization in the Netherlands could be granted, a prospective observational trial was designed, which aimed to analyse the use of trabectedin in STS in a real-world setting.

The original aim of this observational trial was to analyse the use of trabectedin compared to best supportive care (BSC) and derive an incremental cost-effectiveness ratio (ICER) for its use compared to BSC. All patients eligible for trabectedin were also given the option of BSC, but only a few patients opted for BSC, which made it impossible to draw meaningful conclusions from this small number of patients. Instead, as an alternative, a comparison with ifosfamide in retrospective data was sought, as this drug is a treatment option for patients with advanced STS after failure to anthracyclines. Ifosfamide is an alkylating agent and available since the 1980s for the treatment of STSs.

Therefore, this study aims to compare both survival and cost-effectiveness between trabectedin and ifosfamide in the setting of second-line cytostatic treatment of STS in the Netherlands.

## 2. Methods

### 2.1. Patient Selection

In order to facilitate the entry and reimbursement of trabectedin in the Dutch health-care system, a cost-effectiveness analysis was designed to evaluate trabectedin and BSC usage patterns and outcomes in advanced STS in a real-world setting, including data on quality of life and associated utilities. This prospective observational phase IV trial was to provide the Dutch health authority (Zorginstituut Nederland) with sufficient data on the effectiveness and optimal use of trabectedin to ensure a proper evaluation for permanent registry in the Regulation Orphan Drugs. This trial was named ET-D-010-10, with trial registration number NCT01299506. The RECIST 1.1 criteria were used for response evaluation. Quality-of-life data were scored using patient-reported EQ-5D questionnaires. Patients with all subtypes of STS were recruited in this trial if they were eligible for trabectedin, after the failure of anthracyclines and/or ifosfamide, or in case, these patients were unsuited to receive these drugs. The patients in this observational trial were offered treatment with trabectedin or BSC, and the latter could consist of no systemic chemotherapy or other systemic antitumour therapies. Some of the included patients received trabectedin in a different line of therapy than second line, and those patients were not used in the current analysis. All patients were adult and signed an Institutional Review Board approved informed consent form [4].

At the time of the ET-D-010-10 observational trial, no study had yet directly compared the efficacy of trabectedin to BSC. Hence, the choice of treatment was with the patient and local physician, as long as the patient was deemed fit enough to receive chemotherapy. It was intended to include 100 patients, of whom 80 would have received trabectedin and 20 would have chosen BSC. In reality, however, a larger portion of patients wished to be treated with trabectedin (91%) than predicted, and too few patients chose the BSC arm (total 9%: 6% only BSC and 3% received additional or other systemic antitumour therapies). Despite an extension of the trial duration, accrual of the BSC arm was insufficient to be able to perform a viable comparison of the collected data.

To account for the lack of a trial-generated comparator group, it was decided to perform an indirect comparison of the data in the trabectedin arm with data obtained from previous studies. As appropriate data on patients on BSC were not available, an agent active as second-line treatment was sought. These data were obtained from two EORTC clinical trials with ifosfamide in patients with advanced STS, published by Nielsen et al. and van Oosterom et al., hereafter termed “the EORTC trials” [5, 6]. These two trials used the 1979 WHO criteria for response evaluation. According to the 2018 ESMO guideline on STS treatment, after doxorubicin, patients may be treated with ifosfamide, if they did not progress on it previously [1]. Therefore, a second-line setting was chosen for comparing the phase IV ET-D-010-10 data on trabectedin with the EORTC data on ifosfamide.

The efficacy of ifosfamide differs in STS subtypes to a certain extent, but has not been shown to differ as much between subtypes as trabectedin does. Trabectedin has a markedly better efficacy in leiomyosarcoma and liposarcoma subtypes, the so-called L-sarcomas. This difference in efficacy between L-sarcomas and non-L-sarcomas has led to clinical trials which specifically included patients with one of these two subtypes [2]. Due to the prominence of the L-sarcomas in trabectedin clinical research, it was decided to split the study population into two subsets, consisting of L-sarcomas and non-L-sarcomas.

Out of all patients included in the phase IV trial, 54 patients received trabectedin as second-line treatment. The remaining 39 patients received trabectedin as third or higher line of treatment and were excluded from the cost-effectiveness analysis. The drug was prescribed in the accepted regimen of 1.5 mg/m^2^ once every 3 weeks during a 24-hour hospital admission. Due to dose reductions, the average administered trabectedin dose was 1.3 mg/m^2^. From the ifosfamide trials, a total of 50 patients were identified to fit the criterion of second-line treatment. The ifosfamide dosage was 9 g/m^2^ given in 3 consecutive days every 3 weeks (19 patients) [6], or 12 g/m^2^ as a 3-day continuous infusion every 4 weeks (31 patients) [5], together with intravenous Mesna to prevent hemorrhagic cystitis. For the cost-effectiveness analysis, the regimen of 9 g/m^2^ was modelled, as it is current practice in the Netherlands. Based on the EORTC trials, a dose intensity of 95% was implemented.

### 2.2. Survival Analysis

The duration of progression-free survival (PFS) was taken as the time from the first dose of either study drug until disease progression. The latter could be based on radiology findings or in case of trabectedin on clinical evaluation and cessation of treatment due to it. Duration of overall survival (OS) was counted from the day of the first study drug dose until death by any cause. To perform an indirect nonparametric analysis of survival, the Kaplan–Meier method and the logrank test were used. The ECOG performance score was considered to be prognostic for survival, more so than sex or age in patients who require second-line chemotherapy for STS. An ECOG performance score of 0 was classified as low and a score of 1 or 2 as high. A Cox regression analysis was used for multivariate tests, in which ECOG performance score and the drug received were included into the analysis. Survival probabilities at 3 and at 6 months per treatment and group of sarcomas were calculated based on observed progression-free survival, and the number of treatment cycles was noted.

### 2.3. Cost-Effectiveness Analysis

A state-transition model was constructed to estimate healthcare costs and quality-adjusted life years (QALY), separately for the L-sarcoma and the non-L-sarcoma patients. In this model, patients were in a mutually exclusive state of either preprogression survival, postprogression survival (being overall survival (OS) minus preprogression survival), or deceased. The pre- and postprogression average discounted life expectancies (DLEs) were calculated for each treatment. Lifetime costs and QALYs for treatment T, being either trabectedin or ifosfamide, were calculated as follows:

(1) $$\begin{array}{c} {\text{costs}}_{\text{T}}=C_{\text{T}}+C_{\text{preprogression}}\times {\text{DLE}}_{\text{T,preprogression}}+C_{\text{postprogression}}\times {\text{DLE}}_{\text{T,postprogression}},\\ {\text{QALY}}_{\text{T}}=-U_{\text{T}}+U_{\text{preprogression}}\times {\text{DLE}}_{\text{T,preprogression}}+U_{\text{postprogression}}\times {\text{DLE}}_{\text{T,postprogression}}, \end{array}$$

where *C*_T_ is the cost for treatment, such as drug acquisition and administration, and also those due to adverse events and *U*_T_ is the QALY loss due to adverse events. The *C*_preprogression_ and *C*_postprogression_ denote the annual treatment-unrelated costs before and after progression. Similarly, *U*_preprogression_ and *U*_postprogression_ denote the utilities before and after progression. Each of these model parameters is described in more detail below. Subsequently, the incremental cost-effectiveness ratio (ICER) was calculated as follows:

(2) $$\begin{array}{c} \text{ICER}=\frac{{\text{costs}}_{\text{trabectedin}}-{\text{costs}}_{\text{ifosfamide}}}{{\text{QALY}}_{\text{trabectedin}}-{\text{QALY}}_{\text{ifosfamide}}}. \end{array}$$

Consistent with the Dutch guidelines [7], life years (LYs), QALYs, and costs were discounted at 0%, 1.5%, and 4%, respectively. A lifetime horizon was used, and costs are reported in Euros at price level 2018. Other model components are described below. Additional lines of antitumour therapies had not been recorded in the EORTC or ET-D-010-10 trial, and these were not assumed in the cost-effectiveness analysis.

#### 2.3.1. Survival

PFS and OS data of trabectedin and ifosfamide treatments were directly taken from the ET-D-010-10 and EORTC trials, respectively.  Table 1 details the number of patients from each study, as well as baseline characteristics. To estimate average survival times, parametric survival analyses were used, in which all patients were pooled, regardless of treatment. This facilitated extrapolating survival beyond study follow-up and correcting for the (nonsignificant) difference in the ECOG performance score between the prospective trabectedin and retrospective ifosfamide patients. Lognormal distributions were used, based on the Akaike information criterion (data not shown, considered alternative distributions were loglogistic, exponential, gamma, Gompertz, and Weibull distributions).

Table 2 shows the estimated *μ* and *σ* for each treatment in each group of sarcomas for PFS and OS, as well as the associated average survival duration in months and years.

#### 2.3.2. Utilities

Utility values represent the valuation of health, on a scale anchored at 1 for perfect health and 0 for health as poor as deceased. The ET-D-010-10 trial could only provide preprogression utility data, which was scored using the EQ-5D and on average was 0.764. Therefore, EQ-5D utility estimates for patients receiving second-line chemotherapy from the SABINE trial were used. In the SABINE trial, the health-related quality of life was assessed in patients with metastatic sarcoma from North America and Europe, including patients from the Netherlands [8]. Converting the UK tariff to the Dutch tariff resulted in the pre- and postprogression utility score of 0.754 and 0.614, respectively. As the preprogression utility in the SABINE trial was very similar to the utility found in the ET-D-010-10 trial, the usage of the SABINE utilities was considered appropriate. Utilities were assumed equal for L-sarcoma and non-L-sarcoma patients and equal for both trabectedin and ifosfamide treatment groups, except for the disutility caused by adverse events.

#### 2.3.3. Health-Care Costs

Costs for trabectedin and ifosfamide cycles included drug acquisition costs and drug administration costs, as shown in Table 3. Drug administration costs included the costs for hospitalization and blood tests and imaging. The majority of costs for trabectedin cycles consisted of trabectedin acquisition costs, €4,238 out of €5,877 per cycle. For ifosfamide cycles, on the other hand, the 5-day hospitalization formed the largest part of the costs, €2,470 out of €4,474 per cycle. A one-time treatment cost was added to include the cost for insertion of a central venous catheter (CVC), which was mandatory for all patients receiving trabectedin and amounted to €1,015. Nontreatment-related monthly healthcare costs were estimated for patients by extracting these data from the ET-D-010-10 study. These costs were estimated separately from the pre- and postprogression period and assumed equal for L-sarcoma and non-L-sarcoma patients and equal for the trabectedin and ifosfamide treatment groups. During preprogression survival, monthly costs were €284, and during postprogression survival, this rose to €461, as shown in Table 4. Costs were taken from Dutch publicly available sources. Prices were corrected for inflation to obtain 2018 levels.

#### 2.3.4. Adverse Events

Adverse events were scored in the EORTC and wider ET-D-010-10 trials, and the incidence and duration of adverse events were taken directly from these trials, as shown in Table 5. Adverse events were assumed equal for L-sarcoma and non-L-sarcoma patients. Disutility and costs of data per adverse event were taken from the literature and converted to Dutch tariffs and 2018 price levels. In this indirect comparison, trabectedin resulted in more frequent elevation of liver enzymes compared to ifosfamide, whereas ifosfamide gave more neutropenia, with its associated febrile neutropenia. The total QALY loss due to adverse events was 0.00153 for trabectedin and 0.00352 for ifosfamide, with costs of €1,119 and €1,841, respectively.

#### 2.3.5. Sensitivity Analyses

To assess the sensitivity of the model for variations of key parameters, univariate sensitivity analyses were performed and presented in a tornado diagram. The difference in PFS and OS between trabectedin and ifosfamide was varied over the 95% confidence interval (95% CI) in the parametric survival analysis. The other tested variables were increased or decreased by 20%, which included costs of trabectedin, costs of ifosfamide, costs of hospitalization per day, utility preprogression, utility postprogression, and body surface area.

## 3. Results

### 3.1. Patient Characteristics

A total of 54 patients received trabectedin after doxorubicin in the phase IV trial from December 2010 to April 2014, and a total 50 patients were included from the EORTC trials published by Nielsen et al. and van Oosterom et al. The subsets of L-sarcoma and non-L-sarcoma consisted of 57 and 47 patients, respectively, as shown in Table 1.

### 3.2. Survival Analysis

L-Sarcoma patients had a median PFS of 5.2 months on trabectedin, and 2.6 months on ifosfamide, as shown in Table 6. The difference in PFS in this indirect comparison showed a trend favouring trabectedin, but did not reach statistical significance with a *p* value of 0.074. In the multivariate regression, the drug received continued to show a trend with a hazard ratio (HR) of 0.60 (95% CI, 0.33–1.07) and *p* value of 0.086. The median OS for L-sarcoma patients on trabectedin was 11.8 months, and on ifosfamide 8.2 months, also a nonsignificant difference (*p* value, 0.184). For OS, high ECOG performance score at baseline showed an association with reduced survival in both univariate and multivariate tests, with a HR of 1.91 (95% CI, 1.06–3.45) and *p* value 0.032, in the multivariate analysis.

For non-L-sarcoma patients receiving trabectedin, the PFS was 2.0 months, and for patients who received ifosfamide, PFS was 3.3 months, *p* value 0.819. High ECOG performance score was associated with a worse PFS in both univariate test and multivariate test, with a HR of 2.43 (95% CI, 1.16–5.07) and *p* value 0.018 in the latter test. Median OS in this group was 6.0 months for trabectedin and 8.9 months for ifosfamide treatment (*p* value, 0.903). High ECOG performance score was associated with shorter duration of OS, and in the multivariate test, a HR of 2.99 (95% CI, 1.44–6.20), *p* value 0.003.

Patients with an L-sarcoma had a PFS probability at 3 months of 59.5%, and at 6 months of 41.7% when receiving trabectedin, as shown in Table 2. In this group, a mean of 6.1 and median of 6 treatment cycles were given. Patients who had a non-L-sarcoma or who received ifosfamide had shorter survival and received fewer cycles of chemotherapy.

### 3.3. Cost-Effectiveness Analysis

The results from the cost-effectiveness model are shown in Table 7. Results from the parametric survival analysis were consistent with the nonparametric survival analyses. For L-sarcoma patients, trabectedin produced longer PFS and OS than ifosfamide did. For non-L-sarcoma patients, ifosfamide treatment produced longer PFS and OS than trabectedin.

For patients with L-sarcoma, the total discounted costs were €44,879 for trabectedin and €24,797 for ifosfamide. Costs for trabectedin acquisition were higher than for ifosfamide (€31,597 vs. €4,113, respectively), but drug administration costs were lower for trabectedin than ifosfamide (€5,298 vs. €13,380, respectively). The latter difference was due to longer hospitalization needed for ifosfamide cycles. The nontreatment related monthly costs were higher for trabectedin owing to longer survival compared to ifosfamide (€6,866 vs. €5,464, respectively). The costs for adverse events were lower for trabectedin than for ifosfamide (€1,119 vs. €1,841, respectively). These treatments resulted in 1.524 and 1.169 LY gained, respectively, which gives an ICER of €56,000 per LY gained. QALYs were 1,025 for trabectedin and 0.773 for ifosfamide, leading to an ICER of €80,000 per QALY gained.

For patients with a non-L-sarcoma, ifosfamide dominated trabectedin since costs were higher for trabectedin than ifosfamide (€27,497 vs. €22,799, respectively), while effectiveness for trabectedin was worse in terms of LYs (0.754 vs. 1.170, respectively) and in terms of QALYs (0.516 vs. 0.781, respectively).

### 3.4. Sensitivity Analyses

**Figure 1:**
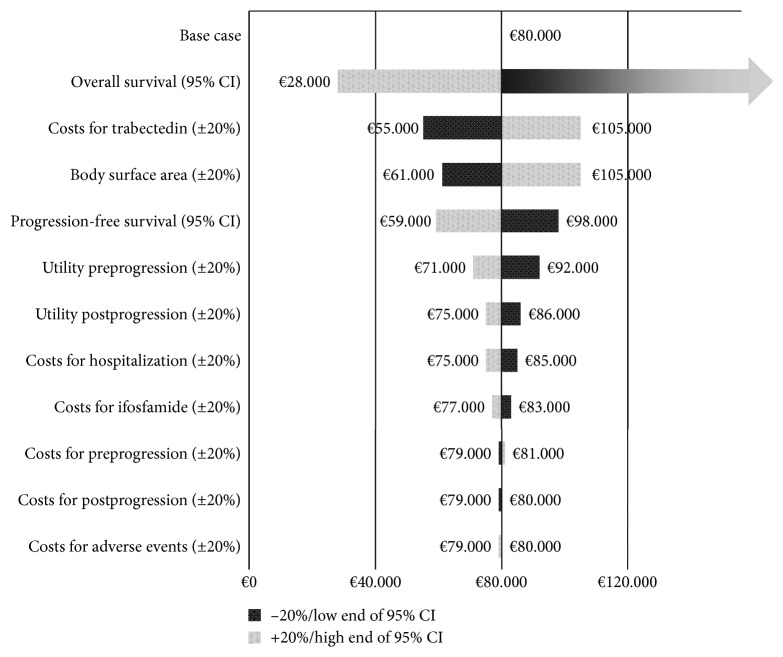
Tornado diagram representing the univariate sensitivity analysis for L-sarcoma, numbers abbreviated to thousands. All variables other than survival were increased (light-shaded bars) or decreased (dark-shaded bars) by 20%. For progression-free survival and overall survival, the 95% confidence interval (95% CI) of the survival difference between trabectedin and ifosfamide was used (low end: light-shaded bars; high end: dark-shaded bars). Note that the bar for the low end of the difference in OS does not stop and no number is given, as ifosfamide dominated trabectedin at that point, with a negative ICER. OS: overall survival; PFS: progression-free survival.

The sensitivity analysis of L-sarcoma showed the ICER to be most affected by the difference in survival between trabectedin and ifosfamide, as shown in Figure 1. This effect was most prominent in OS. The 95% CI of the difference in OS for trabectedin and ifosfamide was −3.6 to 18.4 months, and this meant an overlap of OS duration. This resulted in ICER ranging from €28,000 per QALY gained in favour of trabectedin to ifosfamide being dominant for OS. The ICER across the 95% CI of PFS also varied substantially, but QALYs remained in favour of trabectedin, with the ICER ranging from €59,000 to €98,000 per QALY gained. Another clear influence on ICER variation was the cost of trabectedin, with the ICER ranging from €55,000 to €105,000.

As the base-case analysis showed ifosfamide to dominate trabectedin in patients with non-L-sarcoma, a sensitivity analysis for non-L-sarcoma was not performed.

## 4. Discussion

Trabectedin was shown to be an active drug in the second-line treatment of L-sarcomas (either leiomyosarcoma or liposarcoma). In this nonrandomised comparison, the median survival of patients with L-sarcomas was 2.5 months longer if they received trabectedin instead of ifosfamide, not meeting the criterion for statistical significance (*p*=0.074). In non-L-sarcoma, ifosfamide resulted in longer survival, but the difference was not significant. The cost-effectiveness analysis of trabectedin compared to ifosfamide showed an ICER of €80,000 per QALY gained in case of L-sarcoma. For non-L-sarcoma, ifosfamide dominated trabectedin as ifosfamide costs were lower but survival and QALYs gained higher compared to trabectedin treatment. Survival differences and trabectedin acquisition costs had the strongest impact on the ICERs found. Future changes in trabectedin pricing would alter the ICER. However, given the status of trabectedin as “orphan drug” due to the low incidence of malignancies trabectedin is currently registered for, its price is not expected to change in the foreseeable future.

When this cost-effectiveness analysis was designed, a comparator group was sought that could provide for a sensible comparison to second-line trabectedin. Ifosfamide was chosen as this drug was widely tested in STS, and data for second-line treatment were available at the EORTC. Due to the adverse events and the long hospital admission per treatment cycle, this drug has been used less extensively over last decade, and alternatives are available. In terms of expected antitumour effect, ifosfamide was still considered to represent a realistic comparator group. Additionally, potential alternative data sets would not match the patient population of the trabectedin-treated patients.

This cost-effectiveness study was not a randomised comparison, contrary to the designs of the original ifosfamide studies. To reduce bias, survival was counted from the moment of first drug infusion, not the moment of trial inclusion as in the original trials. This was done to evade a potential bias, wherein the duration of survival of either ifosfamide or trabectedin would have been longer due to effects other than drug effects. Therefore, the difference in survival now reported is accurately reflecting survival following treatment. The EORTC STBSG has used progression-free rates (PFRs) as an indicator whether a drug is active as a second-line agent in STS [22]. Agents considered active have an estimated PFR at 3 months of 39% and at 6 months of 14%. For L-sarcoma, trabectedin showed, by this standard, to be an active drug in this population with a PFR of 59% and 42%, respectively. For non-L-sarcoma, trabectedin was less potent with a PFR at 3 months just below the threshold at 37% and PFR at 6 months at 19%. The PFRs for ifosfamide were above the EORTC STBSG number in both L-sarcoma and non-L-sarcoma.

Several studies have previously investigated the cost-effectiveness of trabectedin in STS compared to other treatments. In a 2011 study by Soini et al., trabectedin was compared to ifosfamide [23]. Trabectedin data were taken from a 2009 randomised trial comparing trabectedin treatment regimen and ifosfamide data from the same studies by van Oosterom and Nielsen used in the current study [5, 6]. All patients on trabectedin had an L-sarcoma, whereas sarcoma subtypes were not clear for patients with ifosfamide. The study found an ICER per LY gained of € 31,590 and € 42,633–47,735 per QALY gained when prescribing trabectedin. These ICERs are lower than in the current study, suggesting better trabectedin cost-effectiveness. The most evident cause for this difference is the higher survival benefit due to ifosfamide in the current study compared to Soini et al. (1.17 LY vs. 0.60 LY, respectively), whereas there were higher costs of ifosfamide treatment in Soini et al. (€13,053–14,286 vs. €7,568, respectively). The difference in survival gained due to ifosfamide, even though these are taken from the same studies, suggests a difference in patient selection between the cost-effectiveness studies.

A 2013 indirect comparison into the cost-effectiveness of doxorubicin-ifosfamide combination vs. trabectedin also showed more QALYs gained at lower health-care costs for doxorubicin-ifosfamide [24]. A pooled patient cohort from four phase II studies of patients receiving trabectedin for advanced STS was used in a 2015 study comparing the cost-effectiveness of trabectedin and the tyrosine kinase inhibitor pazopanib [25]. The HR calculated was 1.11 in favour of pazopanib (with 95% CI of 0.94–1.31). Pazopanib treatment costs were half the cost of trabectedin cycles. As pazopanib is oral medication which is taken without the need for hospital admissions, the majority of patients will prefer pazopanib for that fact alone, regardless of costs. A study comparing pazopanib to placebo in advanced STS patients resulted in an ICER of €77,120 per QALY gained when taking pazopanib treatment, illustrating the high costs of therapies aimed at treating advanced STS [13].

Compared to the 2016 randomised phase III trial by Demetri et al. comparing trabectedin vs. dacarbazine in pretreated metastatic L-sarcoma patients, patients on trabectedin in the current study in a real-life setting had a higher median PFS (4.2 vs. 5.2 months, respectively), whereas OS was slightly lower (12.4 months vs. 11.8 months, respectively) [26]. A possible explanation for the PFS difference is the blinded radiologic evaluation of imaging to assess PFS in the randomised trial. The efficacy of trabectedin vs. dacarbazine showed better PFS for trabectedin but equal OS [26]. Unfortunately, this trial did not include QALY assessments.

A study by Le Cesne ABJYC et al. presented at the 2018 ASCO meeting randomised pretreated advanced STS patients between trabectedin and BSC, giving the comparison originally attempted for this cost-effectiveness analysis [27]. In that trial, trabectedin showed better PFS than BSC for L-sarcomas (5.3 vs. 1.4 months, respectively), but not for non-L-sarcomas (1.8 vs. 1.5 months, respectively). OS did not differ, and this was deemed due to per-protocol crossover to trabectedin after progressive disease on BSC. This trial demonstrates the efficacy of trabectedin for L-sarcomas compared to BSC. The efficacy of trabectedin within the group of L-sarcomas also varies, and it offers the largest benefit in patients with myxoid liposarcoma [28]. The actual size of the antitumour effect in myxoid liposarcoma is blunted in clinical trials as other liposarcoma subtypes, in which trabectedin is less active, and are included in the same trials. The number of patients in this cost-effectiveness analysis was too small to detect any differences between leiomyosarcomas vs. liposarcomas or myxoid liposarcomas vs. other liposarcoma subtypes.

This cost-effectiveness analysis has several limitations, especially since it was not possible to perform the study originally set out to do. The number of included patients was constrained by the number of eligible patients in the ET-D-010-10 and EORTC trials. The criteria for response evaluation were slightly different, but this was considered not to have an impact on the study's conclusion. The nonrandomised nature of the comparison may have introduced bias, especially since the sensitivity analysis showed that the estimated survival difference was the most influential variable in the analysis. The correction for ECOG performance score was enacted to reduce this potential bias. Nevertheless, the *p* values that are reported in the survival analysis disregard the nonrandomized nature of the data.

The use of data on patients treated with ifosfamide did provide a sensible alternative, but those patients were treated some twenty years before the patients who received trabectedin. In those years, experience with safely administering ifosfamide has increased, probably leading to lower adverse event rates than those used in the current study. This may constitute a bias in favour of trabectedin in the study. Other possible explanations for the difference in survival include additional treatment options developed since the ifosfamide trials were performed and advancements in supportive and palliative care.

This study was performed for a Dutch health-care setting with chemotherapy given during hospital admissions. Administrating trabectedin in an outpatient setting using ambulatory pump is also possible [29]. This method of administration would be less costly and will affect the ICER in favour of trabectedin therapy. However, this method is currently not standard in the Netherlands. Obviously, the prices of health-care items will differ in other countries, and the ICER may be different as a result.

## 5. Conclusions

Trabectedin was shown to offer a nonsignificant survival gain compared to ifosfamide for L-sarcoma, and this results in an estimated ICER of €80,000. This ICER is at the top end of what is generally considered acceptable in the Netherlands [30]. As there is a clinically unmet need for antitumour agents in the group of rare malignancies, this threshold may not be the most relevant factor in the decision to continue to prescribe trabectedin to these patients. For non-L-sarcoma, ifosfamide treatment dominated trabectedin.

## Figures and Tables

**Table 1:** Baseline characteristics of study population.

Baseline characteristics of study population		L-Sarcoma number (%)		Non-L-sarcoma number (%)	
		Trabectedin	Ifosfamide	Trabectedin	Ifosfamide
Age at first dose	Mean (SD)	55 (12)	54 (10)	57 (14)	45.3 (14)
Sex	Female	16 (42.1)	9 (47.4)	9 (56.3)	19 (61.3)
	Male	22 (57.9)	10 (52.6)	7 (43.8)	12 (38.7)
ECOG PS	0	18 (47.4)	10 (52.6)	9 (56.3)	8 (25.8)
	1 + 2	20 (52.6)	9 (47.4)	7 (43.8)	23 (74.2)
Study size	ET-D-010-10	38 (100.0)	—	16 (100.0)	—
	Nielsen et al.	—	14 (73.7)	—	17 (54.8)
	Oosterom et al.	—	5 (26.3)	—	14 (45.2)
Drug received	Trabectedin	38 (100.0)	—	16 (100.0)	—
	Ifosfamide	—	19 (100.0)	—	31 (100.0)
Disease status	Local disease	10 (26.3)	1 (5.3)	2 (12.5)	9 (29.0)
	Metastatic disease	28 (73.7)	18 (94.7)	14 (87.5)	22 (71.0)
Tumour histology	Leiomyosarcoma	19 (50.0)	13 (68.4)	—	—
	Liposarcoma	19 (50.0)	6 (31.6)	—	—
	UPS	—		6 (37.5)	4 (19.4)
	Synovial sarcoma	—		5 (31.3)	7 (22.6)
	Neurogenic sarcoma	—		—	4 (12.9)
	Hemangiosarcoma	—		—	3 (9.7)
	Rhabdomyosarcoma	—		—	3 (9.7)
	Others	—		5 (31.3)	8 (25.8)

SD: standard deviation; ECOG-PS: ECOG performance score; UPS: undifferentiated pleomorphic sarcoma.

**Table 2:** Progression-free survival rate age at 3 and at 6 months, the mean and median number of treatment cycles received, the parametric description of survival with the lognormal distribution, and average survival times. The estimated average survival time with the lognormal distribution is calculated by exp (*μ* + *σ*^2^/2).

Progression-free survival		L-Sarcoma		Non-L-sarcoma	
		Trabectedin	Ifosfamide	Trabectedin	Ifosfamide
PFS probability (%)	At 3 months	59.5	47.4	37.5	51.6
	At 6 months	41.7	15.8	18.8	22.6
*N* treatment cycles	Mean	6.1	3.8	3.8	3.4
	Median	6	4	3	3

*Parametric analysis of survival*					
PFS	*μ*	1.50	1.08	1.00	1.20
	*σ*	1.05	1.05	1.04	1.04
Average PFS	In months	7.75	5.07	4.64	5.71
	In years	0.65	0.42	0.39	0.48
OS	*μ*	2.42	2.15	1.77	2.21
	*σ*	1.00	1.00	0.94	0.94
Average OS	In months	18.58	14.17	9.09	14.18
	In years	1.55	1.18	0.76	1.18

PFS: progression-free survival; OS: overall survival.

**Table 3:** Treatment-related costs of trabectedin and ifosfamide, for the average number of treatment cycles (see Table 2).

Treatment-related costs			Trabectedin		Ifosfamide	
Unit	Price	Source	Use	Costs	Use	Costs
Trabectedin 1 mg vial	€1,956	[9]	2.17	€4,238	—	—
Trabectedin 0.25 mg vial	€506	[9]	1.85	€938	—	—
Ifosfamide 2 mg vial	€121	[9]	—	—	8.87	€1,070
Dexamethasone 20 mg vial	€9	[9]	1	€9	—	—
Granisetron 1 mg vial	€4	[9]	2	€8	4	€16
Dexamethasone 8 mg vial	€3	[9]	1	€3	4	€11
Mesna 0.4 mg vial	€9	[9]	—	—	1	€718
Hospitalization per day	€494	[7]	1	€494	5	€2,470
Full laboratory test	€43	[10]	1	€43	1	€43
Haematological test	€18	[10]	0.25	€5	0.25	€5
CT scan	€157	[11]	0.25	€71	0.25	€71
MRI scan	€264	[11]	0.25	€13	0.25	€13
Blood transfusion	€224	[7]	0.25	€56	0.25	€56

*Drug costs per cycle*						
Drug acquisition costs				€5,175		€1,070
Drug administration costs				€702		€3,403
Drug costs, total per cycle				€5,877		€4,474

*One-time treatment costs*						
CVC insertion	€1,015	[11]	1	€1,015	0.30	€305

*Total treatment costs*						
(i) L-Sarcoma				€36,895		€17,081
(ii) Non-L-sarcoma				€23,595		€15,601

Mesna: 2-mercaptoethanesulfonate sodium.

**Table 4:** Nontreatment-related costs per month during preprogression survival and postprogression survival.

Nontreatment-related costs per month			Preprogression survival		Postprogression survival	
Unit	Price	Source	Average use	Cost	Average use	Cost
Hospitalization per day	€494	[7]	0.21	€106	0.48	€236
Full laboratory test	€43	[10]	1.02	€44	1.24	€54
Haematological test	€18	[10]	0.16	€3	0.13	€2
CT scan	€157	[11]	0.31	€49	0.37	€58
MRI scan	€265	[11]	0.01	€2	0.00	€0
Blood transfusion	€224	[7]	0.09	€20	0.00	€0
General practitioner visit	€34	[7]	0.01	€0	0.03	€1
Medical oncologist visit	€102	[7]	0.58	€59	1.08	€110
Nurse	€34	[7]	0.01	€0	0.02	€1
Psychologist	€82	[7]	0.01	€1	0.00	€0
Total costs per period				€284		€461

**Table 5:** Adverse events, frequency, duration, disutility, and costs during trabectedin and ifosfamide treatment.

Adverse events	Frequency of patients (%)		Average duration (days)		Disutility value	Source^*∗*^	QALY loss		Cost per event	Source^†^	Total costs	
	Trabectedin	Ifosfamide	Trabectedin	Ifosfamide			Trabectedin	Ifosfamide			Trabectedin	Ifosfamide
Fatigue and asthenia	2.1	2.2	5.0	5.0	0.216	[12]	0.00006	0.00007	€153	[13]	€3	€3
Nausea	1.0	7.0	10.0	10.0	0.295	[12]	0.00008	0.00056	€1,464	[13]	€15	€120
Vomiting	2.1	5.3	7.5	7.5	0.295	[12]	0.00013	0.00032	€1,464	[13]	€31	€78
Anaemia	5.3	9.6	5.0	5.0	0.098	[14]	0.00007	0.00013	€1,864	[15]	€99	€179
Neutropenia	14.9	39.0	7.5	5.0	0.124	[16]	0.00038	0.00066	€1,329	[15]	€198	€518
Febrile neutropenia	4.3	19.7	12.5	13.0	0.124	[16]	0.00018	0.00087	€2,919	[15]	€6	€575
Thrombocytopenia	13.0	6.1	8.5	1.0	0.089	[17]	0.00027	0.00001	€3,503	[15]	€445	€214
Elevation of liver enzymes	44.7	0.0	3.0	0.0	0.089	#	0.00033	—	€153	[13]	€68	—
Alopecia	0.0	8.3	0.0	36.0	0.094	[16]	—	0.00077	€512	[18]	—	€42
Neurotoxicity	0.0	5.7	1.0	1.0	0.195	[12]	—	0.00003	€1,650	[13]	—	€94
Acute renal failure	0.0	1.8	0.0	16.0	0.124	[19]	—	0.00010	€1,593	[20]	—	€29
Catheter-related infection	2.1	0.1	3.0	0.0	0.161	[17]	0.00003	—	€5,920	[21]	€124	€6
					Total QALY loss		0.00153	0.00352	Total costs		€1,119	€1,841

^*∗*^Translated to Dutch utilities; ^†^translated to 2018 costs in euro's; ^#^assumed similar to thrombocytopenia.

**Table 6:** Nonparametric analysis of survival for L-sarcoma and non-L-sarcoma patients, univariate Kaplan–Meier analysis with median survival in months and logrank test, and multivariate Cox regression. The univariate hazard ratio for age per year increase was 0.99 for all tests.

Nonparametric survival	L-Sarcoma			Non-L-sarcoma		
*Univariate Kaplan–Meier*	Median PFS	95% CI	*p* value	Median PFS	95% CI	*p* value
*Progression-free survival*						
Age		0.96–1.02	0.395		0.97–1.01	0.460
Sex						
(i) Female	3.19	0.00–6.62	0.621	2.89	1.76–4.03	0.931
(ii) Male	4.57	2.43–6.71		2.30	0.81–3.80	
ECOG PS						
(i) 0	3.68	1.08–6.28	0.602	3.22	0.00–6.45	**0.022**
(ii) 1 + 2	3.29	0.00–7.90		1.91	0.46–3.35	
Drug received						
(i) Trabectedin	5.19	3.31–7.07	**0.074**	2.04	1.52–2.55	0.819
(ii) Ifosfamide	2.63	0.43–4.83		3.25	2.33–4.18	
Disease status						
(i) Local	3.29	0.00–7.01	0.740	3.25	0.00–6.80	0.875
(ii) Metastatic	3.94	1.40–6.48		2.43	1.27–4.30	
*Multivariate Cox regression*	HR	95% CI	*p* value	HR	95% CI	*p* value
(i) Drug received	0.60	0.33–1.07	**0.086**	1.35	0.67–2.74	0.403
(ii) ECOG PS	1.11	0.63–1.95	0.715	2.43	1.16–5.07	**0.018**

*Univariate Kaplan–Meier*	Median OS	95% CI	*p* value	Median OS	95% CI	*p* value

*Overall survival*						
Age		0.96–1.02	0.498		0.97–1.01	0.273
Sex						
(i) Female	8.35	2.66–14.0	**0.071**	9.17	5.63–12.7	0.796
(ii) Male	14.85	10.2–19.5		5.55	3.68–7.42	
ECOG PS						
(i) 0	13.41	7.57–19.2	**0.033**	13.77	7.72–19.8	**0.008**
(ii) 1 + 2	8.35	3.92–12.8		5.23	3.64–6.81	
Drug received						
(i) Trabectedin	11.80	7.78–15.8	0.184	5.98	0.70–11.3	0.903
(ii) Ifosfamide	8.22	0.00–21.2		8.94	5.97–11.9	
Disease status						
(i) Local	7.43	2.82–12.0	0.666	11.80	6.30–17.3	0.594
(ii) Metastatic	13.41	7.85–19.0		6.97	2.81–11.1	
*Multivariate Cox regression*	HR	95% CI	*p* value	HR	95% CI	*p* value
(i) Drug received	0.66	0.37–1.18	0.162	1.73	0.85–3.50	0.128
(ii) ECOG PS	1.91	1.06–3.45	**0.032**	2.99	1.44–6.20	**0.003**

PFS: progression-free survival; OS: overall survival; 95% CI: 95% confidence interval; ECOG PS: ECOG performance score; HR: hazard ratio.

**Table 7:** Estimated average costs and effectiveness, comparing trabectedin and ifosfamide in advanced L-sarcoma and non-L-sarcoma.

Cost-effectiveness model	L-Sarcoma			Non-L-sarcoma		
	Trabectedin	Ifosfamide	Difference	Trabectedin	Ifosfamide	Difference
*Costs (all discounted)*	€44,879	€24,797	€20,082	€27,497	€22,799	€4,698
(i) Drug acquisition	€31,597	€4,113	€27,484	€19,407	€3,660	€15,747
(ii) Drug administration	€5,298	€13,380	−€8,082	€3,646	€11,941	− €8,295
(iii) Nonrelated costs	€6,866	€5,464	€1,402	€3,325	€5,357	− €2,032
(iv) Adverse events costs	€1,119	€1,841	−€722	€1,119	€1,841	− €722

*Effectiveness*						
(i) QALYs, discounted	1.025	0.773	0.251	0.516	0.781	−0.265
(ii) Preprogression LYs, undiscounted	0.646	0.423	0.223	0.386	0.476	−0.090
(iii) Postprogression LYs, undiscounted	0.902	0.758	0.144	0.371	0.694	−0.323
*Cost-effectiveness ratios*						

(i) Costs per LY gained	**€56,000**			Ifosfamide dominant		
(ii) Costs per QALY gained	**€80,000**			Ifosfamide dominant		

QALYs: quality-adjusted life years; LY: life years.

## Data Availability

The raw data used to support the findings of this study are available from the corresponding author upon request.
